# Changes in endothelial function during educational hospitalization and the contributor to improvement of endothelial function in type 2 diabetes mellitus

**DOI:** 10.1038/s41598-020-72341-8

**Published:** 2020-09-21

**Authors:** Yukiko Goshima, Yosuke Okada, Keiichi Torimoto, Yoshihisa Fujino, Yoshiya Tanaka

**Affiliations:** 1 First Department of Internal Medicine, School of Medicine, University of Occupational and Environmental Health Japan, Kitakyushu, Japan; 2 Department of Endocrinology and Metabolism, Toranomon Hospital, Tokyo, Japan; 3 Department of Environmental Epidemiology, Institute of Industrial Ecological Sciences, University of Occupational and Environmental Health Japan, Kitakyushu, Japan

**Keywords:** Atherosclerosis, Type 2 diabetes

## Abstract

Only a few reports have examined vascular endothelial function before and after educational hospitalization and the factors that affect it in patients with type 2 diabetes mellitus (T2DM). The aim of this study was to assess vascular endothelial function before and after educational hospitalization and identify factors that affect it. In 65 patients with T2DM who underwent peripheral arterial tonometry (EndoPAT) before and after hospitalization, vascular endothelial function (reactive hyperemia index [RHI]), glucose metabolism, lipid metabolism, and blood pressure were assessed before and after hospitalization. The primary endpoint was hospitalization-induced changes in vascular endothelial function. Educational hospitalization significantly improved the natural logarithmically scaled RHI (L_RHI) from 0.555 ± 0.212 to 0.625 ± 0.245 (p = 0.012). Multivariable logistic regression analysis identified hypoglycemia during hospitalization as the single factor that significantly altered vascular endothelial function (p = 0.019). The odds of achieving normal vascular endothelial function were 0.08 times lower (95% confidence interval, 0.01–0.67) for each episode of hypoglycemia. Furthermore, multivariable analysis identified hypoglycemia during hospitalization as the single factor that worsened L_RHI. Our study showed that educational hospitalization of patients with T2DM improved vascular endothelial function, and that the development of hypoglycemic episodes had a significant negative impact on normalization of vascular endothelial function.

## Introduction

Type 2 diabetes mellitus (T2DM) is associated with vascular endothelial dysfunction even at the early stage, and atherosclerosis plays a major role in the development of macrovascular disease. For example, the risk of cerebral infarction, ischemic heart disease, and myocardial infarction is 2–6 times higher in patients with T2DM^1,2^. Vascular endothelial dysfunction is related to the occurrence and reoccurrence of cardiovascular events; therefore, assessment of vascular endothelial function is useful in the evaluation of arteriosclerosis and risk assessment of cardiovascular disease^3^. Several factors are thought to contribute to vascular endothelial dysfunction in T2DM, including hyperglycemia, hyperinsulinemia with insulin resistance, and hypoglycemia. We have assessed the relevance of vascular endothelial function and hypoglycemia in T2DM^4–10^. In particular, oxidative stress induced by postprandial hyperglycemia and fluctuations in blood glucose levels is associated with vascular endothelial dysfunction^4,9^.

The STOP-NIDDM (Study TO Prevent Non Insulin Dependent Diabetes Mellitus) Trial research group^11^ reported that treatment of impaired glucose tolerance with acarbose is associated with reduced risk of cardiovascular events. Furthermore, management of dyslipidemia, quitting smoking, exercise, and use of angiotensin-converting enzyme (ACE) inhibitors and angiotensin receptor blockers (ARBs) may improve vascular endothelial function and prevent cardiovascular diseases^3^.

In-patient diabetes education programs improve blood glucose control^12^, and the combination of diet and exercise also improve lipid metabolism and blood pressure. Although improvement of various metabolic abnormalities may subsequently improve vascular endothelial function; little is known about the outcome of in-patient diabetes education, especially with regard to vascular endothelial dysfunction. Moreover, it is not clear which factor(s) related to vascular endothelial function are affected by such programs. The present study was designed to document the changes in endothelial function in T2DM patients after receiving in-patient diabetes education and the variables associated with these changes.

## Methods

### Study patients

Among patients with T2DM admitted to the Hospital of the University of Occupational and Environmental Health and Wakamatsu Hospital of the University of Occupational and Environmental Health between September 2010 and May 2017, 65 were selected for this study. Their selection was based on fulfillment of the following inclusion criteria: (1) peripheral arterial tonometry (EndoPAT) was performed as part of the routine clinical care and patient education, before and after hospitalization, (2) age > 20 years, (3) no diabetic ketosis or nonketotic hyperosmolar coma during hospitalization, and (4) no infection or other acute diseases during hospitalization.

This study was conducted according to the tenets of the Helsinki Declaration and current ethical codes. The study protocol was approved by the ethical committee of the University of Occupational and Environmental Health (Trial registration: H27-186, Registered 25 Dec 2015), and an opt-out method of informed consent was approved by the committee.

### Study design

This was a retrospective study based on data available in the medical records of patients admitted to the hospital. Patients with T2DM underwent blood tests (for assessment of glucose metabolism, lipid metabolism, and blood pressure) and EndoPAT (reactive hyperemia index [RHI] for evaluation of vascular endothelial function) at the time of both admission and discharge for inpatient diabetes education. Vascular endothelial function was evaluated using peripheral arterial tonometry (EndoPAT2000; Itamar Medical, Caesarea, Israel). Glucose metabolism was assessed by calculating the average blood glucose and M-values of blood glucose level from data recorded by the self-monitoring blood glucose (SMBG) technique at the following time points: before breakfast, before lunch, before dinner, and 120 min after dinner^13^. An episode of hypoglycemia on SMBG was defined as a blood glucose level < 70 mg/dL. Lipid metabolism was assessed by measurement of triglycerides (TGs), high-density lipoprotein cholesterol (HDL-C), and low-density lipoprotein cholesterol (LDL-C).

The primary endpoint was a change in vascular endothelial function between at discharge relative to that at admission. The secondary endpoints were factors associated with improvement of vascular endothelial function.

### Intervention

Both facilities provided diabetes education following similar programs. All patients received a 2-week education program including lectures from doctors, pharmacists, and nurses using brochures, slides, and videos. As part of diet therapy, salt intake was limited to 6 g and energy intake was set at 25 to 30 kcal/kg of the ideal body weight (60% carbohydrate, 15–20% protein, and 20–25% fat). The patients individually received at least one session of nutrition education from a dietitian during their hospitalization. Exercise therapy was performed for roughly 15 to 30 min after a meal, two to three times a day. A calorie counter was used and the target was set at 8,000 to 10,000 steps per day. Pharmacotherapy was selected in consideration of body type, insulin secretory ability, complications, and adherence, and physicians, pharmacists, and nurses explained the drugs to be used to the patients. In addition, education regarding diabetic complications, hypoglycemia, and target blood glucose level was also provided. Improvement in body weight and various metabolic abnormalities due to the education program and diet and exercise therapies can be expected to lead to behavioral changes after discharge.

### Noninvasive vascular function test

The method used for the digital measurement of vascular function was based on peripheral arterial tonometry as described in detail previously^14^. Briefly, after a 30-min acclimatization period in a room controlled for temperature and light with the patient in the fasting state, the baseline pulse amplitude was recorded during a 5-min period before the induction of local ischemia. Ischemia was induced by placing the blood pressure cuff on the upper arm, while the opposite arm served as a control. The peripheral arterial tonometry probes were placed on one finger of each hand. After 5 min, the blood pressure cuff was inflated to 60 mmHg above the systolic pressure or to 200 mmHg for 5 min and then deflated to induce reactive hyperemia. As a measure of reactive hyperemia, RHI was calculated as the ratio of the average amplitude of the peripheral arterial tonometry signal over 1 min beginning 1.5 min after cuff deflation (control arm, A; occluded arm, C) divided by the average amplitude of the peripheral arterial tonometry signal over the 2.5-min time period before cuff inflation (baseline) (control arm, B; occluded arm, D). Thus, RHI = (C/D)/(A/B) × baseline correction. Because RHI has a heteroscedastic error structure, we used the natural logarithm transformation in all analyses.

### Statistical analysis

Data are expressed as mean ± standard deviation. The Wilcoxon signed-rank test was used to assess differences in the natural logarithmically scaled RHI (L_RHI), blood glucose, lipid, and blood pressure between admission and discharge. The cutoff value of vascular endothelial disorder was set as L_RHI < 0.51 according to previous studies^14–17^. We categorized patients with L_RHI ≥ 0.51 at the time of discharge into the normal vascular endothelial function group, whereas the others were classified into the low vascular endothelial function group. The odds ratios (ORs) for the normal vascular endothelial function at the time of discharge were estimated using univariate and multivariate logistic regression. The model included sex, age, body mass index (BMI), duration of diabetes mellitus, use of ACE inhibitors/ARBs/statins, current smoking, glucose metabolism, blood pressure, and lipid metabolism to investigate the relationship between L_RHI and hypoglycemia. We selected a similar indicator from which multicollinearity may occur for each factor, and statin use was excluded as it produced multicollinearity in the preliminary analysis. We used univariable and multivariable linear regression analyses to estimate regression coefficients for the change in vascular endothelial function (ΔL_RHI). The model was fed with data of sex, age, BMI, duration of diabetes mellitus, change in blood glucose metabolism, change in blood pressure, and change in lipid metabolism. We selected a similar indicator from which multicollinearity may occur for each factor and examined it with two models because the Δaverage and ΔM-value were found to be multicollinear in the preliminary analysis. All statistical tests were performed with SPSS Statistical Software 25.0 (SPSS Inc., Chicago, IL), and the results were considered significant when the *p* value was < 0.05.

## Results

### Clinical characteristics

Table 1 shows the clinical characteristics of the participating patients. The 65-patient study population comprised 29 men and 36 women with a mean age of 60.5 ± 12.6 years (range, 22–84 years). The patients were mildly obese, with a mean BMI of 26.7 ± 5.0 kg/m^2^. The mean duration of diabetes mellitus was 8.7 ± 11.1 years (range, 0–47 years). Of the patients, 28 (43%) were smokers and 3 (5%) had history of cardiovascular diseases. The mean systolic blood pressure was 129.7 ± 17.5 mmHg (range, 93–171 mmHg), and the mean diastolic blood pressure was 76.5 ± 12.5 mmHg (range, 49–112 mmHg). The mean fasting plasma glucose level was 158.2 ± 49.6 mg/dL (range, 88–355 mg/dL), and the HbA1c value was 9.5% ± 2.3% (range, 6.3–17.0%). A comparison of patients with normal vascular endothelial function (high L_RHI group, L_RHI ≥ 0.51) and those with vascular endothelial dysfunction (low L_RHI group, L_RHI < 0.51) on admission is presented in Supplemental Table 1.

**Table 1:** Baseline characteristics (n = 65).

	Mean ± SD	Min	Max
Age, years	60.5 ± 12.6	22	84
Male sex, n (%)	29 (45)		
BMI (kg/m^2^)	26.7 ± 5.0	18.2	45.7
Duration, years	8.7 ± 11.1	0	47
Smoking, n (%)	28 (43)		
Neuropathy, n (%)	31 (48)		
Retinopathy, n (%)	16 (25)		
Nephropathy, n (%)	21 (32)		
CVD, n (%)	3 (5)		
Systolic blood pressure, mmHg	129.7 ± 17.5	93	171
Diastolic blood pressure, mmHg	76.5 ± 12.5	49	112
HbA1c, (%)	9.5 ± 2.3	6.3	17.0
Fasting plasma glucose, mg/dL	158.2 ± 49.6	88	355
IRI, μU/mL (n = 63)	7.2 ± 5.9	1.3	38.9
u-CPR, μg/day (n = 63)	99.2 ± 65.0	0.2	355.6

Data are mean ± SD, n, or n (%).

SD, standard deviation; BMI, body mass index; HbA1c, hemoglobin A1c; CVD, cardiovascular disease; IRI, immunoreactive insulin; u-CPR, urinary C-peptide immunoreactivity.

### Intervention

In this study, 50 of the 65 patients used oral hypoglycemic drugs. Specifically, the number of patients using sulfonylurea (SU) decreased, whereas the number of patients using pioglitazone, metformin (MET), α-glucosidase inhibitor (αGI), dipeptidyl peptidase-4 inhibitor, glucagon-like peptide-1 (GLP-1) analogue, insulin, and sodium glucose cotransporter 2 inhibitor (SGLT2-I) increased from admission to discharge (Table 2). No changes were observed in treatments with lipid-lowering agents and antihypertensive agents during hospitalization.

**Table 2:** Changes in glucose-lowering medications during hospitalization (n = 65).

	Admission	Discharge
Day of evaluation after admission	4.2 ± 2.1	18.8 ± 3.0
Diet, n (%)	15 (23)	0 (0)
Sulfonylurea, n (%)	37 (57)	26 (40)
Pioglitazone, n (%)	6 (9)	11 (17)
Metformin, n (%)	16 (25)	26 (40)
α-Glucosidase inhibitor, n (%)	3 (5)	16 (25)
Dipeptidyl peptidase-4 inhibitor, n (%)	28 (43)	34 (52)
Glucagon-like peptide-1 analog, n (%)	0 (0)	5 (8)
Insulin, n (%)	2 (3)	9 (14)
Sodium glucose cotransporter 2 inhibitor, n (%)	1 (2)	30 (46)

Data are n (%).

### Changes in various clinical parameters

Table 3 shows the clinical parameters at admission and discharge. Compared with those at admission, blood glucose levels before each meal and at 2 h after dinner were significantly improved at discharge (p < 0.001, each). Moreover, the average blood glucose level changed from 177 ± 50.4 to 129 ± 21.8 mg/dL, and the M-value improved significantly from 19.0 ± 22.6 to 6.0 ± 4.3 mg/dL (p < 0.001, each).

**Table 3:** L_RHI, blood glucose, blood pressure, and serum lipid parameters at admission and discharge (n = 65).

	Admission	Discharge	p-value
L_RHI	0.555 ± 0.212	0.625 ± 0.245	0.012
Blood glucose level before breakfast, mg/dL	149.4 ± 38.3	112.4 ± 25.2	< 0.001
Blood glucose level before lunch, mg/dL	190.6 ± 74.0	125.1 ± 33.3	< 0.001
Blood glucose level before dinner, mg/dL	150.9 ± 55.6	119.5 ± 31.2	< 0.001
Blood glucose level after dinner, mg/dL	218.0 ± 68.0	160.1 ± 37.4	< 0.001
Average glucose, mg/dL	177 ± 50.4	129 ± 21.8	< 0.001
M-value, mg/dL	19.0 ± 22.6	6.0 ± 4.3	< 0.001
Systolic blood pressure, mmHg	130 ± 17.5	122 ± 15.6	< 0.001
Diastolic blood pressure, mmHg	76 ± 12.5	73 ± 10.7	0.006
Triglycerides, mg/dL (n = 62)	155 ± 74.3	111 ± 38.7	< 0.001
LDL-cholesterol, mg/dL (n = 62)	120 ± 34.9	96 ± 27.7	< 0.001
HDL-cholesterol, mg/dL (n = 62)	47.9 ± 13.5	46.2 ± 10.8	0.121
LDL-to-HDL ratio (n = 62)	2.68 ± 0.95	2.19 ± 0.80	< 0.001

Data are mean ± standard deviation, n, or n (%).

L_RHI, natural logarithmically scaled reactive hyperemia index; LDL, low-density lipoprotein; HDL, high-density lipoprotein.

However, 15 of the 65 patients (23%) developed hypoglycemia, and their antidiabetic therapy were as follows: SU + MET + DPP4-I + SGLT2-I in 4 patients, SU + DPP4-I + SGLT2-I in 2 patients, SU + MET + SGLT2-I in 1 patient, SU + αGI + DPP4-I + SGLT2-I in 1 patient, MET + DPP4-I in 1 patient, MET + insulin in 1 patient, MET + DPP4-I + SGLT2-I in 1 patient, MET + αGI + DPP4-I in 1 patient, pioglitazone + insulin in 1 patient, MET alone in 1 patient, and SGLT2-I alone in 1 patient. Both systolic and diastolic blood pressure levels were significantly lower at discharge (p < 0.001, p = 0.006).

### Factors associated with normal vascular endothelial function at discharge

Vascular endothelial function was evaluated after admission on mean day 4.2 ± 2.1 of admission and on mean day 18.8 ± 3.0 at discharge. The natural logarithmically scaled RHI (L_RHI) significantly increased from 0.555 ± 0.212 at admission to 0.625 ± 0.245 after discharge (p = 0.012) (Table 3). Previous studies proposed L_RHI of < 0.51 as the cutoff value for vascular endothelial disorder^13–16^. Accordingly, we divided the patients into two groups: the normal vascular endothelial function group (high L_RHI group, L_RHI ≥ 0.51) and the low vascular endothelial function group (low L_RHI group, L_RHI < 0.51) at discharge. The high L_RHI group included 63% (41 patients), whereas the low L_RHI group included 37% (24 patients).

A comparison of patients with normal vascular endothelial function and those with vascular endothelial dysfunction at the time of discharge is presented in Supplemental Table 2. Univariable and multivariable logistic regression analyses were performed considering patients with normal vascular endothelial function at discharge (Table 4). The analysis identified hypoglycemia to be significantly associated with high L_RHI (p = 0.019). The odds for normal vascular endothelial function were 0.08 times lower with each 1-point increase in hypoglycemia (95% confidence interval, 0.01–0.67).

**Table 4:** Clinical markers associated with L_RHI ≥ 0.51 at discharge.

	Univariable logistic regression		Multivariable logistic regression	
	OR (95% CI)	p	OR (95% CI)	p
Sex, male/female	0.92 (0.33–2.57)	0.880	0.56 (0.20–4.11)	0.890
Age, per year	1.03 (0.99–1.08)	0.124	1.65 (0.75–3.62)	0.213
BMI, per kg/m^2^	0.93 (0.83–1.03)	0.158	0.91 (0.36–2.27)	0.841
Duration, years	1.02 (0.98–1.08)	0.377		
ACE-I or ARBs, yes/no	2.13 (0.72–6.88)	0.185	3.74 (0.65–21.5)	0.138
Statin, yes/no	1.30 (0.47–3.75)	0.614		
Current smoking, yes/no	1.07 (0.85–1.37)	0.577	0.91 (0.19–4.36)	0.909
Average glucose, mg/dL	1.02 (0.99–1.05)	0.153	0.85 (0.37–1.92)	0.691
M-value, mg/dL	1.02 (0.90–1.16)	0.775	1.40 (0.59–3.34)	0.448
Hypoglycemia, yes/no	0.19 (0.05–0.64)	0.010	0.08 (0.01–0.67)	0.019
SBP, mmHg	1.03 (0.99–1.07)	0.093	0.93 (0.38–2.29)	0.877
DBP, mmHg	1.02 (0.98–1.07)	0.440		
Triglycerides, mg/dL	0.99 (0.97–1.00)	0.076		
LDL-cholesterol, mg/dL	1.01 (0.99–1.03)	0.537		
HDL-cholesterol, mg/dL	1.07 (1.01–1.15)	0.036		
LDL to HDL ratio	0.80 (0.46–1.36)	0.411	0.85 (0.41–1.78)	0.670

L_RHI, natural logarithmic scaled reactive hyperemia index; OR, odds ratio; CI, confidence interval; BMI, body mass index; ACE-I, angiotensin-converting enzyme inhibitor; ARB, angiotensin receptor blocker; SBP, systolic blood pressure; DBP, diastolic blood pressure; LDL, low-density lipoprotein; HDL, high-density lipoprotein.

**Figure 1:**
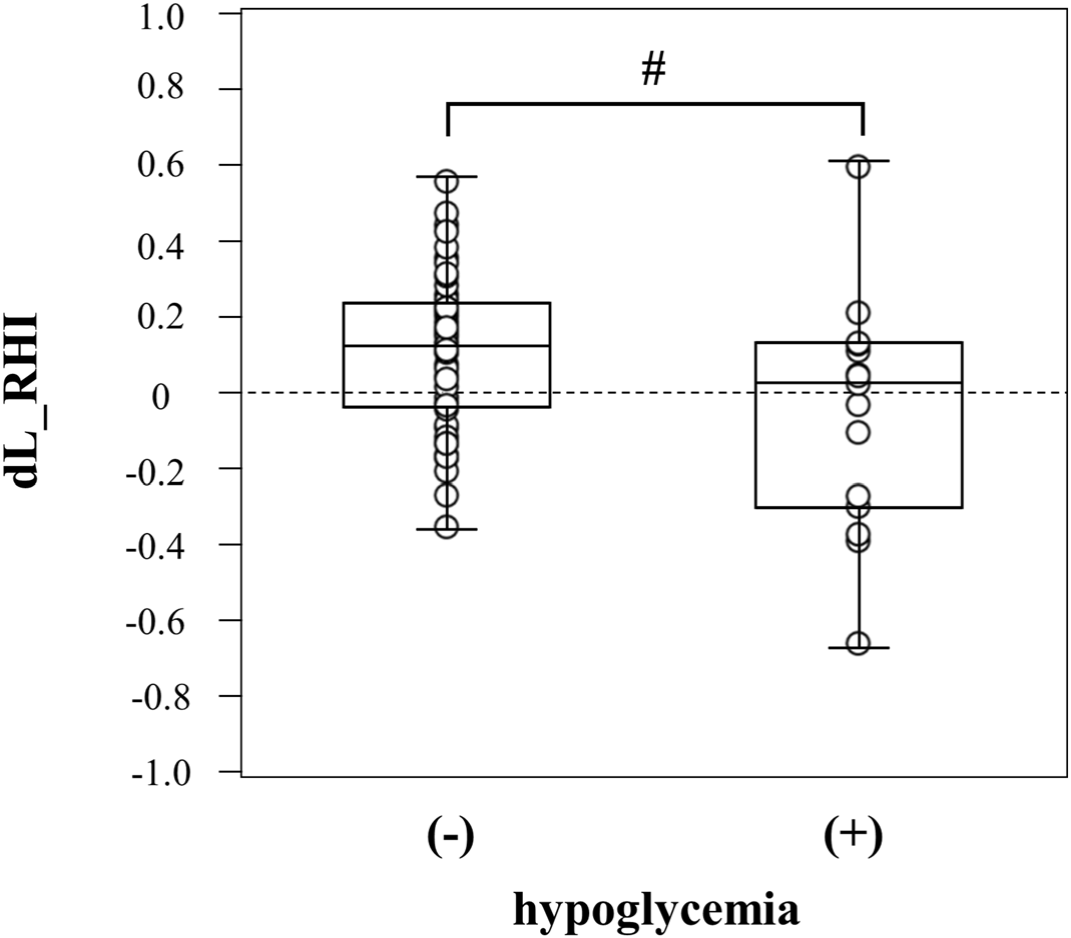
Changes in endothelial function in patients with and without hypoglycemia. ^#^p < 0.05. L_RHI, natural logarithmically scaled reactive hyperemia index.

Univariable and multivariable linear regression analyses were performed to estimate the regression coefficients for change in vascular endothelial function. The results identified hypoglycemia during educational hospitalization as a significant determinant of changes in L_RHI (Supplemental Table 3). In fact, the change in L_RHI (ΔL_RHI) was significantly lower (− 0.053 ± 0.308 vs. 0.108 ± 0.205, p < 0.05) in patients who developed hypoglycemia (Fig. 1).

Next, the patients were divided into four groups for comparison: 1) the consistently normal group (N group), 2) the abnormal RHI during admission to normal at discharge group (A → N group), 3) the persistently abnormal group (A group), and 4) the normal RHI during admission to abnormal at discharge group (N → A group). The incidence of hypoglycemia showed significant differences between the four groups (P = 0.017). The highest incidence noted was 0.5 in the N → A group (Supplemental Figure 1).

Finally, the blood glucose profiles at the time of discharge were compared between patients with and without hypoglycemia (Supplemental Table 4). In patients with hypoglycemia, the pre-breakfast, pre-dinner, and mean blood glucose levels were significantly lower.

## Discussion

The main findings of the present study were: (i) hospitalization of patients for education about T2DM, including fine tuning medications and provision of diabetes-related educational program, improved glucose metabolism, lipid metabolism, blood pressure, and vascular endothelial function, and that (ii) hypoglycemia was associated with vascular endothelial dysfunction. Educational programs for patients with T2DM are known to correct lifestyle and enhance treatment mainly based on blood glucose control^18,19^. However, only a few studies have analyzed the effects of in-hospital T2DM-related educational programs on vascular endothelial function. The findings of the present study suggest that although in-hospital educational programs improve vascular endothelial function by correcting various metabolic abnormalities, hypoglycemia is a factor that hinders such improvement.

In the present study, diet and exercise (for 15–30 min per session, 2–3 sessions per day) therapies were initiated on admission, and oral hypoglycemic drugs were selected based on clinical conditions and glucose dynamics. These measures resulted in improvement in glucose dynamics. With respect to the effects of dietary changes on vascular endothelial function, Kondo et al.^20^ reported that improvement in postprandial hyperglycemia contributes to improved vascular endothelial function. Another report indicated that physical exercise increases the production of nitric oxide (NO) and improves vascular endothelial function through increased physiological activation of NO^21^. Furthermore, with regard to the association between glucose metabolism and vascular endothelial function, a study using the glucose clamp technique reported that fluctuations in blood glucose levels are associated with vascular endothelial dysfunction^22^. Our group has also reported that fluctuations in blood glucose levels, postprandial hyperglycemia, and hypoglycemia are associated with vascular endothelial dysfunction in diabetic patients^4^.

In the present study, the application of strict diet and exercise therapies improved both lipid metabolism and blood pressure. In patients with hypertension, vascular endothelial dysfunction is associated with increase in blood pressure, probably resulting from NO deactivation (which promotes vasodilation), which is caused by overproduction of reactive oxygen species; with subsequent decrease in bioavailable NO due to decreased production and release of NO; and with the increase in vasodilatory substances, such as reactive oxygen species and arachidonate metabolites^23^. The average daily sodium intake in Japanese people, as estimated from urine sodium excretion, is reportedly 12.1 g in men and 10.2 g in women^24^. It is possible that during the hospitalization, the consumption of sodium-restricted diet (6 g sodium) leads to correction of excessive sodium intake-induced hypertension, which in turn, improves vascular endothelial function. In patients with dyslipidemia, the decrease in the production and release of endothelium-derived relaxing factors causes vascular endothelial dysfunction. Experimental studies have shown that LDL inhibits endothelium-dependent relaxation^25^, and it seems that hypercholesterolemic state appears to have a similar effect^26^. As described above, blood glucose, blood pressure, and lipids have separate effects on vascular endothelial function; therefore, a comprehensive treatment is important in maintaining vascular endothelial function. Because these conditions can be treated during educational admission to the hospital, in-patient diabetes education seems to improve vascular endothelial function.

Our results suggest that the absence of hypoglycemic episodes contributes to normalization of vascular endothelial function. Previous studies demonstrated that strict blood glucose control increases the likelihood of development of hypoglycemic episodes^27^, which is an independent risk factor for cardiovascular events^28^. Hypoglycemia is considered to cause vascular endothelial dysfunction due to the associated increases in reactive oxygen species, catecholamines^29^, and proinflammatory cytokines^30^. We have assessed the relevance of hypoglycemia and glycemic variability in T2DM^4,31–34^. In particular, we reported the association between hypoglycemia and vascular endothelial function in a cross-sectional study of actual clinical cases^4^, the present study on changes before and after hospitalization therapy showed that the prevention of hypoglycemia is important for treatment that aims to achieve favorable vascular endothelial function. In our previous study using continuous glucose monitoring (CGM), we reported that the risk of hypoglycemia is higher with a lower mean blood glucose level and bigger fluctuations in blood glucose levels^33^. The present study also revealed that the mean blood glucose level was lower in patients with hypoglycemia. It seems necessary to set a higher target for blood glucose control in patients at a risk of developing hypoglycemia. In addition, the risk of hypoglycemia is known to be higher with bigger fluctuations in blood glucose levels^33^ and fluctuations in blood glucose levels have been reported to be associated with vascular endothelial function^4^. To determine the effects of fluctuations in blood glucose levels due to hypoglycemia on vascular endothelial function, detailed assessment of glucose dynamics using CGM is necessary. This issue needs to be investigated in the future.

Previous studies showed that correction of individual metabolic abnormalities improves vascular endothelial function. However, our study is the first to show that vascular endothelial function is improved by comprehensive management through in-hospital education program and that the extent of such improvement is attenuated in patients with hypoglycemia. In this regard, the Steno 2^35^ and JDOIT3^36^ studies that used cardiovascular events as the outcome of treatment of T2DM showed that comprehensive management of blood pressure, lipids, and blood glucose may result in the prevention of cardiovascular events. However, another large-scale study; the ACCORD Study^37^ showed that a similar comprehensive management strategy failed to prevent cardiovascular events and that serious hypoglycemia increased the risk of death in diabetic patients^38^. In our study, we showed for the first time that improvement in vascular endothelial function could be hindered by hypoglycemia even after in-hospital education program. Hence, while it is important to control hyperglycemia in diabetics through medications, it is equally important to prevent hypoglycemia as a consequence of use of such medications.

The present study identified BMI and the use of ACE inhibitors or ARBs as factors affecting normal vascular endothelial function on admission. In obesity, vascular endothelial dysfunction is reported to be associated with decreased NO production due to uncoupling of the endothelial nitric oxide synthase and enhanced NO degradation which in turn is attributable to increased nicotinamide adenine dinucleotide phosphate oxidase^39^. In fact, it has been reported that RHI is lower with a higher BMI in patients with concurrent obesity/metabolic syndrome^40^. Furthermore, a previous study reported that ACE inhibitors and ARBs improve vascular endothelial function by decreasing C-reactive protein levels^41^. The present study also revealed that BMI and the use of ACE inhibitors or ARBs were associated with vascular endothelial function, as indicated in previous reports.

The present study has several limitations. First, it was a retrospective study conducted on a small population of patients admitted to two clinical facilities. Consequently, it was difficult to perform detailed subgroup analyses based on oral hypoglycemic drugs, lipid metabolism, blood pressure, and other factors. In the present study, the effects of blood pressure, blood glucose levels, and age, among other factors, on vascular endothelial function could not be assessed and confounding factors including drugs could not be controlled for. A large-scale multicenter study needs to be conducted in the future to evaluate the separate effect of each individual metabolic abnormality on the observed improvement in vascular endothelial function induced by in-hospital education program. Second, glucose dynamics were assessed using blood glucose levels measured at four time points. Because vascular endothelial function is associated with fluctuations in blood glucose levels and postprandial hyperglycemia as measured by continuous glucose monitoring (CGM), studies involving detailed assessment of glucose dynamics using CGM may be needed in the future. Third, because vascular endothelial function was assessed only at two time points of admission and discharge in the present study, it is unclear whether vascular endothelial function was assessed at appropriate time points. However, previous reports^6,7,42^ have indicated that changes in vascular endothelial function can be observed within a few hours to a few days after therapeutic interventions. In the present study, the post-treatment assessment was performed at average 18.8 days after the therapeutic interventions. This period appears to be appropriate for examining changes in vascular endothelial function. Fourth, ambulatory blood pressure monitoring (ABPM) was not performed in the present study. Because vascular endothelial function has already been reported to be associated with fluctuations in blood pressure as measured using 24-h ABPM^43^, assessment of data from ABPM was necessary to assess the effects of blood pressure on vascular endothelial function. In addition, the present study identified high systolic blood pressure on admission as a factor contributing to normal vascular endothelial function. This may be because the present study included patients with well-controlled blood pressure, as demonstrated by a mean blood pressure of 130 mmHg, and many patients with hypertension used ACE inhibitors or ARBs. The effects of these factors may not have been excluded.

## Conclusions

The present study showed that educational hospitalization of patients with T2DM improves vascular endothelial function, and that prevention of hypoglycemic episodes enhances the normalization of endothelial function. To our knowledge, this is the first study that has dissected the effects of educational hospitalization on T2DM-related vascular endothelial function. Our results indicate that educational hospitalization improves vascular endothelial function by correcting various metabolic abnormalities and that hypoglycemia hinders improvement in vascular endothelial function.

## Supplementary information

Supplementary information.

## Data Availability

All data generated or analysed during this study are included in this published article.
