# Supplementary material for: Changes in endothelial function during educational hospitalization and the contributor to improvement of endothelial function in type 2 diabetes mellitus
Source: Sci Rep. 2020 Sep 21;10:15384. doi: 10.1038/s41598-020-72341-8 (PMC7506545; doi:10.1038/s41598-020-72341-8)
Supplement: Supplementary file 1 — Supplementary information. [file 41598_2020_72341_MOESM1_ESM.docx]

**Changes in endothelial function during educational hospitalization and the contributor to improvement of endothelial function in type 2 diabetes mellitus**

Yukiko Goshima^1,2^, Yosuke Okada^1^, Keiichi Torimoto^1^, Yoshihisa Fujino^3^, and Yoshiya Tanaka^1^*

^1^First Department of Internal Medicine, School of Medicine, University of Occupational and Environmental Health Japan, Kitakyushu, Japan

^2^Department of Endocrinology and Metabolism, Toranomon Hospital, Tokyo, Japan

^3^Department of Environmental Epidemiology, Institute of Industrial Ecological Sciences, University of Occupational and Environmental Health, Kitakyushu, Japan

**Supplementary information**

Supplemental Table 1

Supplemental Table 2

Supplemental Table 3

Supplemental Table 4

Supplemental Figure 1

**Supplemental Table 1. Participants characteristics between normal vascular endothelial function group (L_RHI ≥0.51) and low vascular endothelial function group (L_RHI <0.51) at hospitalization.**

|  | L_RHI ≥0.51 | L_RHI <0.51 | p-Value |
| --- | --- | --- | --- |
| N | 33 | 32 |  |
| Male, n (%) | 15 (45.5) | 14 (43.8) | 0.891 |
| Age, per year | 62.9±11.2 | 58.1±13.7 | 0.217 |
| BMI, per kg/m^2^ | 25.4±4.3 | 28.1±5.3 | 0.049 |
| Duration, years | 7.6±10.6 | 9.7±11.7 | 0.146 |
| ACE inhibitors or ARBs, n (%) | 15 (45.5) | 8 (25.0) | 0.085 |
| Statin, n (%) | 16 (48.5) | 11 (34.4) | 0.248 |
| Current smoking, n (%) | 15 (45.5) | 13 (40.6) | 0.694 |
| Average glucose, mg/dL | 187.0±53.2 | 167.1±45.9 | 0.089 |
| M-value, mg/dL | 22.9±24.3 | 14.9±20.4 | 0.135 |
| Systolic blood pressure mmHg | 136.1±19.6 | 123.1±12.2 | 0.003 |
| Diastolic blood pressure mmHg | 78.7±14.4 | 74.1±9.8 | 0.162 |
| Triglycerides, mg/dL | 150.1±82.8 | 156.1±63.8 | 0.408 |
| LDL-cholesterol, mg/dL | 120.0±30.4 | 118.6±39.8 | 0.555 |
| HDL-cholesterol, mg/dL | 50.5±15.1 | 46.3±12.2 | 0.299 |

L_RHI, natural logarithmic scaled reactive hyperemia index; BMI, body mass index; ACE, angiotensin-converting enzyme; ARB, angiotensin receptor blocker; LDL, low-density lipoprotein; HDL, high-density lipoprotein.

**Supplemental Table 2. Participants characteristics between normal vascular endothelial function group (L_RHI ≥0.51) and low vascular endothelial function group (L_RHI <0.51) at discharge.**

|  | L_RHI ≥0.51 | L_RHI <0.51 | p-Value |
| --- | --- | --- | --- |
| N | 41 | 24 |  |
| Male, n (%) | 18 (43.9) | 11 (45.8) | 0.881 |
| Age, per year | 62.4±12.0 | 57.3±13.4 | 0.213 |
| BMI, per kg/m^2^ | 25.3±4.2 | 27.0±5.5 | 0.392 |
| Duration, years | 9.6±12.8 | 7.0±7.6 | 0.956 |
| ACE inhibitors or ARBs, n (%) | 17 (41.4) | 6 (25.0) | 0.180 |
| Statin, n (%) | 18 (43.9) | 9 (37.5) | 0.613 |
| Current smoking, n (%) | 18 (43.9) | 10 (41.7) | 0.861 |
| Average glucose, mg/dL | 132.2±20.3 | 124.2±23.5 | 0.091 |
| M-value, mg/dL | 6.1±3.93 | 5.8±5.0 | 0.568 |
| Hypoglycemia, n (%) | 5 (12.2) | 10 (41.7) | 0.006 |
| Systolic blood pressure mmHg | 124.2±16.2 | 117.4±13.6 | 0.165 |
| Diastolic blood pressure mmHg | 73.4±11.5 | 71.3±9.2 | 0.605 |
| Triglycerides, mg/dL | 104.9±35.9 | 123.8±42.0 | 0.133 |
| LDL-cholesterol, mg/dL | 97.7±28.9 | 93.1±25.4 | 0.508 |
| HDL-cholesterol, mg/dL | 48.3±11.3 | 42.0±8.7 | 0.035 |

L_RHI, natural logarithmic scaled reactive hyperemia index; BMI, body mass index; ACE, angiotensin-converting enzyme; ARB, angiotensin receptor blocker; LDL, low-density lipoprotein; HDL, high-density lipoprotein.

**Supplemental Table 3. Linear multivariable analysis with ΔL_RHI as the dependent variable**

|  | Univariable linear regression | | | |  | Multivariable linear regression | | | | | | |
| --- | --- | --- | --- | --- | --- | --- | --- | --- | --- | --- | --- | --- |
|  |  |  |  |  |  | Model 1 | | |  | Model 2 | | |
|  |  | β | SE | P |  | β | SE | P |  | β | SE | P |
| Intercept |  |  |  |  |  | 0.203 | 0.314 | 0.520 |  | 0.159 | 0.306 | 0.604 |
| Sex, male/female |  | 0.032 | 0.060 | 0.593 |  | 0.010 | 0.070 | 0.885 |  | 0.006 | 0.069 | 0.932 |
| Age, per year |  | 0.0005 | 0.0020 | 0.848 |  | -0.0001 | 0.00030 | 0.978 |  | 0.0004 | 0.0030 | 0.865 |
| BMI, per kg/m^2^ |  | -0.005 | 0.006 | 0.445 |  | -0.002 | 0.008 | 0.978 |  | -0.001 | 0.008 | 0.604 |
| Duration, per year |  | 0.0004 | 0.003 | 0.875 |  |  |  |  |  |  |  |  |
| Δaverage, mg/dL |  | 0.0005 | 0.001 | 0.489 |  | 0.001 | 0.001 | 0.366 |  |  |  |  |
| ΔM-value, mg/dL |  | -0.001 | 0.001 | 0.392 |  |  |  |  |  | -0.002 | 0.001 | 0.172 |
| Hypoglycemia, yes/no |  | -0.162 | 0.068 | 0.021 |  | -0.170 | 0.077 | 0.033 |  | -0.187 | 0.078 | 0.021 |
| ΔSBP, mmHg |  | -0.003 | 0.002 | 0.055 |  | -0.002 | 0.002 | 0.191 |  | -0.002 | 0.002 | 0.224 |
| ΔDBP, mmHg |  | -0.003 | 0.002 | 0.211 |  |  |  |  |  |  |  |  |
| ΔTriglycerides, mg/dL |  | 0.0010 | 0.0005 | 0.042 |  |  |  |  |  |  |  |  |
| ΔLDL-C, mg/dL |  | 0.001 | 0.001 | 0.148 |  |  |  |  |  |  |  |  |
| ΔHDL-C, mg/dL |  | 0.001 | 0.004 | 0.760 |  |  |  |  |  |  |  |  |
| ΔLDL to HDL ratio |  | 0.046 | 0.038 | 0.697 |  | 0.013 | 0.042 | 0.750 |  | 0.011 | 0.042 | 0.784 |
| R^2^ |  |  |  |  |  | 0.165 | | |  | 0.181 | | |

We used univariable and multivariable linear regression analysis to estimate regression coefficients for change in vascular endothelial function (ΔL_RHI). The model fed into sex, age, BMI, duration of diabetes mellitus, change of blood glucose metabolism, change of blood pressure and change of lipid metabolism. We selected one of the similar indicators from which multicollinearity may occur for each factor and examined in two models because of Δaverage and ΔM-value were found to be multicollinear in preliminary analysis.

β, regression coefficient; L_RHI, natural logarithmically scaled reactive hyperemia index; SE, standard error; CI, confidence interval.

**Supplemental Table 4. Blood glucose parameters at discharge with and without hypoglycemia.**

|  | Hypoglycemia (-) | Hypoglycemia (+) | p-Value |
| --- | --- | --- | --- |
| N | 50 | 15 |  |
| Blood glucose level before breakfast, mg/dL | 117.1±24.5 | 96.7±21.6 | 0.001 |
| Blood glucose level before lunch, mg/dL | 128.4±33.2 | 114.2±32.2 | 0.137 |
| Blood glucose level before dinner, mg/dL | 124.4±31.7 | 103.1±23.3 | 0.023 |
| Blood glucose level after dinner, mg/dL | 162.1±34.8 | 153.5±45.6 | 0.139 |
| Average glucose, mg/dL | 133.0±19.7 | 116.9±24.2 | 0.006 |
| M value, mg/dL | 5.8±3.8 | 6.9±5.5 | 0.249 |

Data are mean±standard deviation or n.

**Supplemental Figure 1**


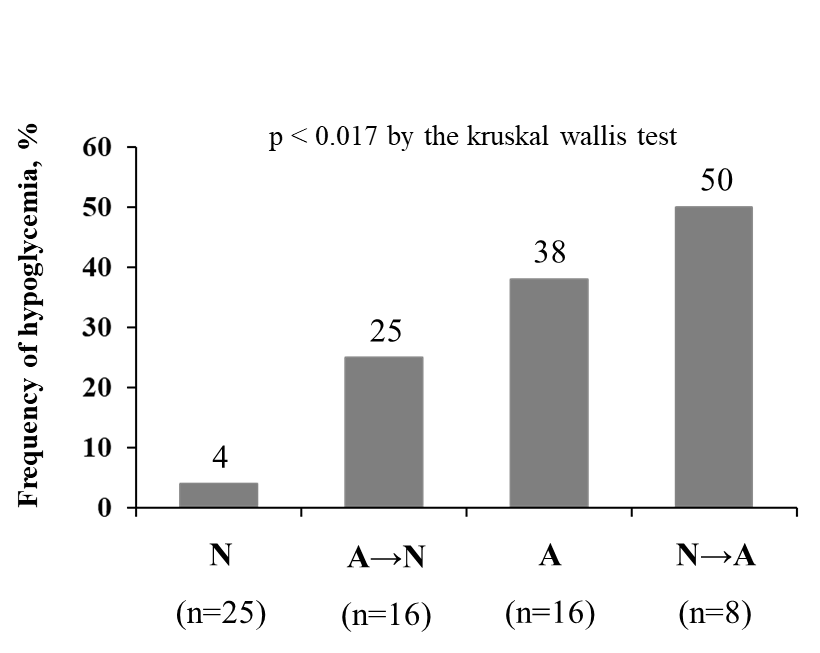


**Figure legend**

Supplemental Figure 1. Relationship between the conditions of endothelial functions and hypoglycemia.

The patients were divided into four groups: 1) the consistently normal group (N group), 2) the abnormal endothelial function during admission to normal at discharge group (A→N group), 3) the persistently abnormal group (A group), and 4) the normal endothelial function during admission to abnormal at discharge group (N→A group).

Abnormal endothelial function: L_RHI <0.51.

L_RHI, natural logarithmically scaled reactive hyperemia index.
